# Characterization of Lignin Structures in *Phyllostachys edulis* (Moso Bamboo) at Different Ages

**DOI:** 10.3390/polym12010187

**Published:** 2020-01-10

**Authors:** Yikui Zhu, Jiawei Huang, Kaili Wang, Bo Wang, Shaolong Sun, Xinchun Lin, Lili Song, Aimin Wu, Huiling Li

**Affiliations:** 1 State Key Laboratory for Conservation and Utilization of Subtropical Agro-Bioresources, South China Agricultural University, Guangzhou 510642, China; ZhuYKWerid@163.com (Y.Z.); jiaweihuangkawy@gmail.com (J.H.); wangkl_china@163.com (K.W.); 2 Guangdong Key Laboratory for Innovative Development and Utilization of Forest Plant Germplasm, College of Forestry and Landscape Architectures, South China Agricultural University, Guangzhou 510642, China; 3 The Key Laboratory of Plant Molecular Breeding of Guangdong Province, College of Agriculture, South China Agricultural University, Guangzhou 510642, China; bowang@scau.edu.cn; 4 College of Natural Resources and Environment, South China Agricultural University, Guangzhou 510642, China; sunshaolong328@scau.edu.cn; 5 State Key Laboratory of Subtropical Silviculture, Zhejiang A&F University, Lin’an 311300, China; lxc@zafu.edu.cn (X.L.); lilisong@zafu.edu.cn (L.S.)

**Keywords:** *Phyllostachys edulis*, lignin structures, molecular weight, S:G ratio, NMR

## Abstract

Bamboo is a gramineous plant widely distributed in China and has great prospects. Normally, local people cut bamboo culm at first year for paper milling or at six years for construction. Understanding lignin changes in bamboo with aging is necessary for better exploring the application of bamboo at different ages and can also promote the application of bamboo more effectively. Based on the previous study, the chemical structure and the lignin content of bamboo at different ages were further explored by FT-IR, GPC, NMR and other chemical methods in this paper. Results showed that the lignin structures of bamboo at different ages were similar with three monomers of S, G and H, but the molecular weight increased with age. Quantitative structure estimation further confirmed that S-type lignin content and S/G ratio of bamboo lignin constantly increased with age.

## 1. Introduction

Bamboo belong to the Gramineae plant group, which is widely distributed in Asian countries [1]. Due to the global shortage of forest resources, bamboo is considered the best plant species to replace wood because of its rapid growth, high tensile strength, good toughness and ductility [2]. More attention has focused on the broad prospects of bamboo as an energy plant besides the traditional pulp industry in recent years, such as packaging industry [3,4]. The application of bamboo is mainly related to its secondary cell wall [5]. Compared with the secondary cell wall of wood, bamboo has a more obviously layered structure. Unlike wood, the material properties of bamboo will not be affected by its high-speed growth. Moreover, characteristics of bamboo such as high mechanical strength, good toughness and ductility has attracted growing attention from scholars [6]. However, there are few studies on the changes of bamboo cell wall in the process of lignification with age, which restricts the application of bamboo as an energy plant to some extent. The main components of the secondary cell wall of *Phyllostachys pubescens* (bamboo) are cellulose, hemicellulose and lignin. The lignification of bamboo is a dynamic process of deposition of lignin in the secondary cell wall [7]. Therefore, understanding the changes of lignin can give an insight into the cell wall changes during bamboo lignification.

Lignin is an amorphous polymer which contains three different cinnamyl alcohol monomers: coniferyl, sinapyl, and *p*-coumaryl alcohols, each of which leads to a corresponding type of lignin unit named guaiacyl, syringyl, and p-hydroxyphenyl, respectively, linked by C–C bonds and ether bonds [8]. As the second most abundant natural polymer, lignin accounts for about 30% of the organic carbon in the biosphere, and it is composed of aromatic monomers [9]. In the process of plant growth, lignin is polymerized in vivo via an enzyme-mediated dehydrogenation polymerization (lignification), resulting in a cross-linked amorphous material with both C–C and ether bonds [10]. Moreover, lignin plays an important role in strengthening cell-to-cell connections, increasing cell wall strength and resisting pathogens during subsequent growth [11]. In the field of biological research, modern biotechnology provides precise manipulation techniques for the biosynthesis of lignin, which can alter the lignin structure in plants by up- or down-regulating specific genes. Therefore, future research on lignin should combine the synergy between biology and chemistry. Nowadays, lignin is widely used in the production of aromatic chemical products instead of petroleum-based raw materials. The main trends are the separation of three main aromatic monomers by depolymerization of lignin: benzene, toluene, and xylene, then subsequent synthesis of other chemical products [9,12,13].

Although the application prospect of lignin is very clear, it now has more obstacles to its efficient utilization. The extraction methods of lignin can be divided into two categories: (1) use of a neutral solvent to dissolve and extract a portion of the lignin while retaining the carbohydrates; (2) an enzymatic hydrolysis method to degrade the carbohydrates to retain the residual lignin [9]. Although lignin separation methods have been studied for many years, there are still many scholars working on new techniques to explore new lignin separation approaches with higher yields and less structural damage [14]. Monomers extracted from lignin are not suitable for direct preparation of compounds [9]. The physical and chemical properties of lignin produced by content changes of nonpolymeric alcohols in different plant sources are quite different, which is one of the obstacles in industrialization. In the process of plant lignification, the formation of lignin monomer from cross-linking to each other to form lignin macromolecules adds further complexity to the lignin structure [15]. Therefore, the structural complexity of lignin of different species is also an important constraint.

Bamboo, which is one of the rapid growth lignocellulosic biomass species, is considered to be a suitable raw material for the lignin industry due to its characteristics of wide distribution and high lignin content [16]. Large-scale plantings of bamboo usually takes no more than six years to reach harvestable size in China. The study of the lignification of bamboo has mostly focused on the growth pattern and anatomical structure of bamboo [17]. However, the chemical quantification of lignin has not been explored at different bamboo ages. In general, bamboo with lower lignin content is favorable for the paper making and bioenergy industry applications, while bamboo with higher lignin content is more suitable for use in the building and furniture industries. Therefore, a clear understanding of the changes in bamboo lignin with age can further guide the follow-up bamboo industry. The purpose of this paper is to investigate the changes in chemical structure and content of lignin of bamboo with ages (1–6 years old). Based on the previous research [17,18], the analysis of bamboo lignin in different years was carried out by Nuclear Magnetic Resonance (NMR), Fourier Transform infrared spectroscopy (FT-IR), Gel Permeation Chromatography (GPC), and changes in lignin chemical structures during the whole lignification process was observed visually.

## 2. Materials and Methods

### 2.1. Materials

1–6 year old bamboo used in this study were collected from Hangzhou, Zhejiang Province, China. Prior to the experiments, the bamboo was decorticated, then sieved to about 40-mesh size. The obtained particles were oven-dried at 55 °C to a constant weight. The composition analysis (cellulose, hemicellulose and lignin) of bamboo was performed according to the procedure established by National Renewable Energy Laboratory [19].

### 2.2. Milled Wood Lignin (MWL) Isolation and Purification

The lignin of 1–6 year(s) old bamboo were isolated according to the previous literature [20]. First, 20 g of bamboo samples from different years after extraction and drying were subjected to ball milling for 2.5 h by a planetary ball mill (DECO-PBM-V, Deco Co., Ltd., Changsha, Hunan, China) at 300 rpm using zirconia balls of approximately 1 cm in diameter. After ball milling, the solid is re-baked for use. The dried solid was mixed with 96% dioxane (*w*/*v*, 1:20) and stirred for 2.5 h at 100 °C. After the reaction, the solid–liquid separation was carried out using a sand core funnel. The separated liquid was concentrated by a rotary evaporator (Rotavapor^®^ R-300, BUCHI, Flawil, Switzerland), and then dropped into 3 volumes of 95% ethanol for 24 h to remove excess hemicellulose. After the reaction, centrifugation was carried out, and the liquid fraction was concentrated again. The concentrated liquid was dropped into 10 volumes of acid water (pH = 2, hydrochloric acid) to precipitate the desired lignin. The precipitated solid was freeze-dried to obtain the required lignin products.

### 2.3. Determination and Structural Characterization of Bamboo Lignin

FT-IR spectra of the prepared 1–6 year(s) old bamboo lignin samples were recorded on a spectrophotometer (Tensor 27, Bruker Optics, Karlsruhe, Germany) in the range of 1800–800 cm^−1^ using a KBr disc containing finely ground samples (1%) [21]. Lignin acetylation was carried out with reference to relevant literature [22], and the molecular weight of acetylated lignin was determined by GPC. In short, 1 mg acetylated lignin was dissolved in 1 mL of tetrahydrofuran (≥99.0%, HPLC, Sigma, Oliver Township, MI, American), and then filtered through an organic phase filter and measured by GPC (Nexera UHPLC/HPLC System Shimadzu, Kyoto, Japan) [21]. Experiments were repeated three times to obtain the average values. NMR spectra (^13^C NMR and 2D-HSQC spectra) of the lignin samples were obtained on a 600 MHz Bruker Avance (Tensor 27, Bruker Optics, Karlsruhe, Germany). The ^13^C NMR analysis conditions were: 100 mg lignin dissolved in 0.5 mL DMSO-d_6_, sampling time was 1.35 s, relaxation time was 1.5 s, scanned 3000 times; 2D-HSQC analysis conditions were: 60 mg lignin dissolved in 0.5 mL DMSO-D_6_, the sampling time was 0.17 s, the relaxation time was 1.5 s, sampled 128 times, and the scanning time was 6 h [23].

## 3. Results and Discussion

### 3.1. Composition Analysis of 1–6 Year(s) Old Bamboo

**Figure 1:**
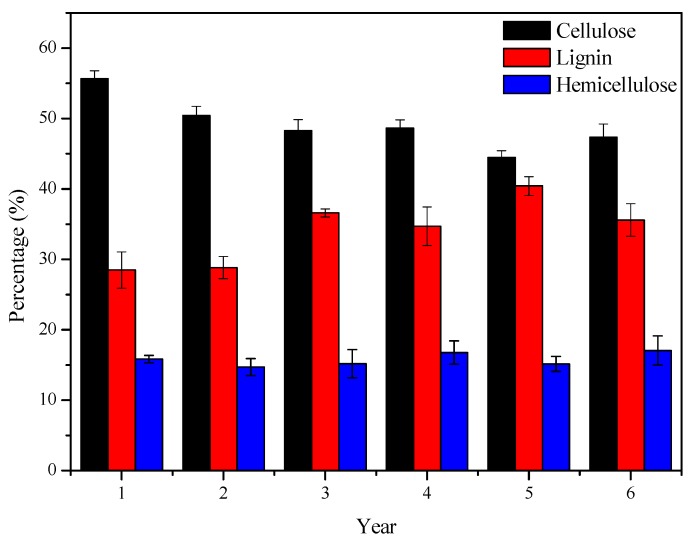
1–6 years old bamboo composition analysis.

The changes in contents of cellulose, hemicellulose and lignin were examined during the lignification of bamboo. Compared with hemicellulose, the contents of lignin and cellulose varied more obviously with ages (Figure 1). The lignification of bamboo is the generation of lignin. Our study showed that a growing trend (26–36%) of lignin content could be observed from 1–6 years old bamboo. However, the relative content of cellulose decreased (55–45%) with stable hemicellulose amount. The earliest phase of the growth of bamboo (between one and two years) is the rapid accumulation of biomass. After this, the stem grows rapidly to the maximum height, followed by the increase in strength and accumulation of dry mass. The processes of lignification and cell wall thickening begin after three years [24]. It can be seen from Figure 1 that the relative content of lignin in one and two-year old bamboo is almost the same, and the lignin contents of bamboo in three to six years old are significantly higher than those in the former. This phenomenon is consistent with the results about the chemical composition of bamboo during the lignification process [25]. The above results indicate that the growth rates of lignin, hemicellulose and cellulose are in the following order: lignin > hemicellulose > cellulose.

### 3.2. FT-IR Spectra

**Figure 2:**
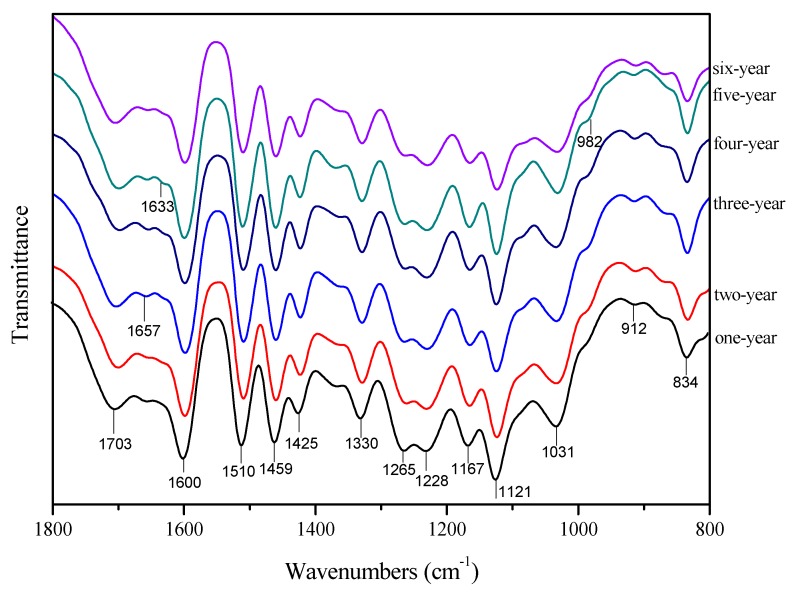
FT-IR spectra of 1–6 years old bamboo lignin.

In order to investigate the changes of lignin structure during bamboo lignification, FT-IR was conducted to analyze the structure of lignin extracted from different annual bamboo, and the results are shown in Figure 2. Characteristic brands of lignin skeleton and fractions were assigned according to data from the literature. The absorbance at 1703 cm^−1^ corresponds to the conjugated carbonyl (C=O) [26]. Brands at 1600 cm^−1^, 1501 cm^−1^, and 1425 cm^−1^ are the characteristic peaks of the benzene ring structure, which proved that the bamboo lignin has a typical benzene ring structure [27]. The absorption peak at 1459 cm^−1^ is the C–H deformation vibration associated with the benzene ring [28]. The three absorption peaks are the characteristic peaks of lignin skeleton structure, their strengths are almost the same in each year, which proves that the lignin skeleton does not change significantly during the lignification of the bamboo. The peak at 1330 cm^−1^ is the absorption peak of the syringyl group and the condensed guaiacyl group. The absorption peak at 1265 cm^−1^ corresponds to the C=O stretching vibration in the guaiacyl ring, while the peak at 1228 cm^−1^ corresponds to the vibration of lignin C–C, C–O, C=O. The peak at 1167 cm^−1^ is attributed to the ester bond, which indicated that the bamboo lignin monomers are linked by ester bonds [29]. Peaks at 834 cm^−1^, 1121 cm^−1^ and 1167 cm^−1^ were attributed to the three monomers of lignin, respectively, suggesting that the bamboo lignin contained three lignin monomers of S, G and H. FT-IR results indicate that the basic skeleton of lignin remains consistent in the process of bamboo lignification. Three lignin monomers of S, G and H exist in all bamboo lignin samples. FT-IR spectra of 1–6 years old bamboo lignin samples behave such that the absorption peak position and relative intensities are basically the same, which proves that the structure of bamboo lignin in different years had no major modifications occurred during lignification. Therefore, in order to better explore these changes, more analysis is necessary.

### 3.3. Molecular Weight Analysis

Molecular weight is one of the important properties of polymers. The weight average (Mw) and number average (Mn) molecular weight of lignin obtained from one to six years-old bamboo were determined by GPC (Table 1). The Mw of 1–6 years-old bamboo lignin increased from 4586 to 5823 with age. Moreover, the molecular weight polydispersity coefficient PD (Mw/Mn) of lignin increased with the degree of lignification, which indicates that the molecular weight distribution of the obtained samples is wider. The change in molecular weight during lignification deepened, further indicating that the older the bamboo, the more suitable it is for the preparation of lignin-based composites than the lower-grade bamboo [30].

### 3.4. ^13^C NMR Spectra

**Figure 3:**
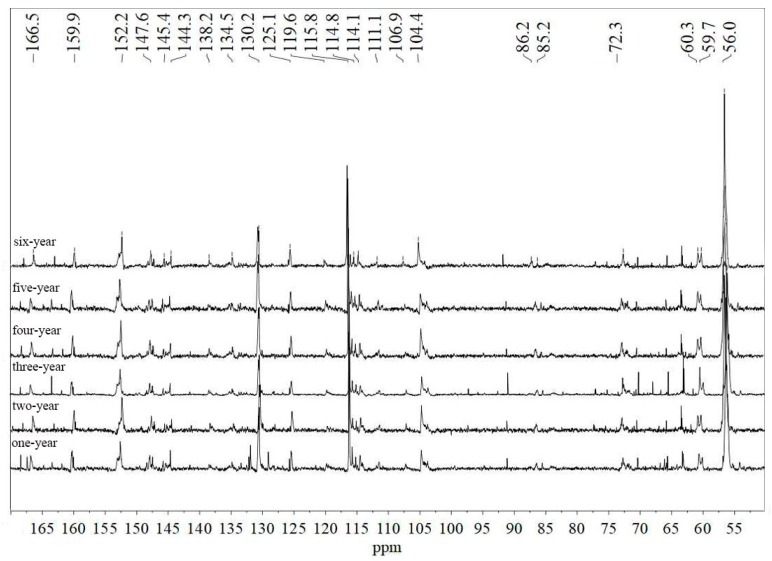
^13^C NMR spectra of 1–6 year-old bamboo lignins.

To further characterize the lignin structures with ages, different annual bamboo lignin were measured by ^13^C NMR (Figure 3). Peak assignments are made by comparison with the data reported in the literature [31,32]. Overall, the lignin peaks of bamboo in different years are roughly the same in addition to peak intensity, which is consistent with the results of FT-IR. The characteristic peaks for coumarin esters appear at δ166.5 ppm, δ159.9 ppm, δ144.3 ppm, δ130.2 ppm, δ125.1 ppm, δ115.9 ppm, and δ114.8 ppm, corresponding to C9, C4, C7, C2/6, C1, C3/5 and C8. The signal peaks of the C8 junction appeared in the six-year old bamboo lignin, indicating that the synthesis of lignin side chains occurred in the γ position during the lignification of bamboo. Characteristic signal peaks of S-type lignin unit appear at δ152.3 ppm, δ147.6 ppm, δ138.2 ppm, δ134.5 ppm, and δ106.9 ppm, respectively, corresponding to etherified C3/C5, non-etherified C3/C5, etherified C4, etherified C1, non-etherified C2/C6 connection location. The characteristic peaks of the G-type lignin unit appear at δ145.1 ppm, δ119.5 ppm, and δ111.2 ppm, corresponding to non-etherified C4, C6 (etherified and non-etherified), and C2 (etherified and non-etherified) position. Peaks of H-type lignin unit appear at δ130.2 ppm, which corresponds to the C2/C6 linkage. Both of the above signal peaks can be found in all bamboo lignin carbon spectra, which proves that the bamboo lignin is composed of three lignin monomers (S, G, and H). This phenomenon is consistent with the FT-IR results mentioned before. The results of ^13^C NMR confirmed that the structure of lignin monomer and the substitution joint position did not change much during the lignification of bamboo.

### 3.5. 2D-HSQC NMR Spectra

**Figure 4:**
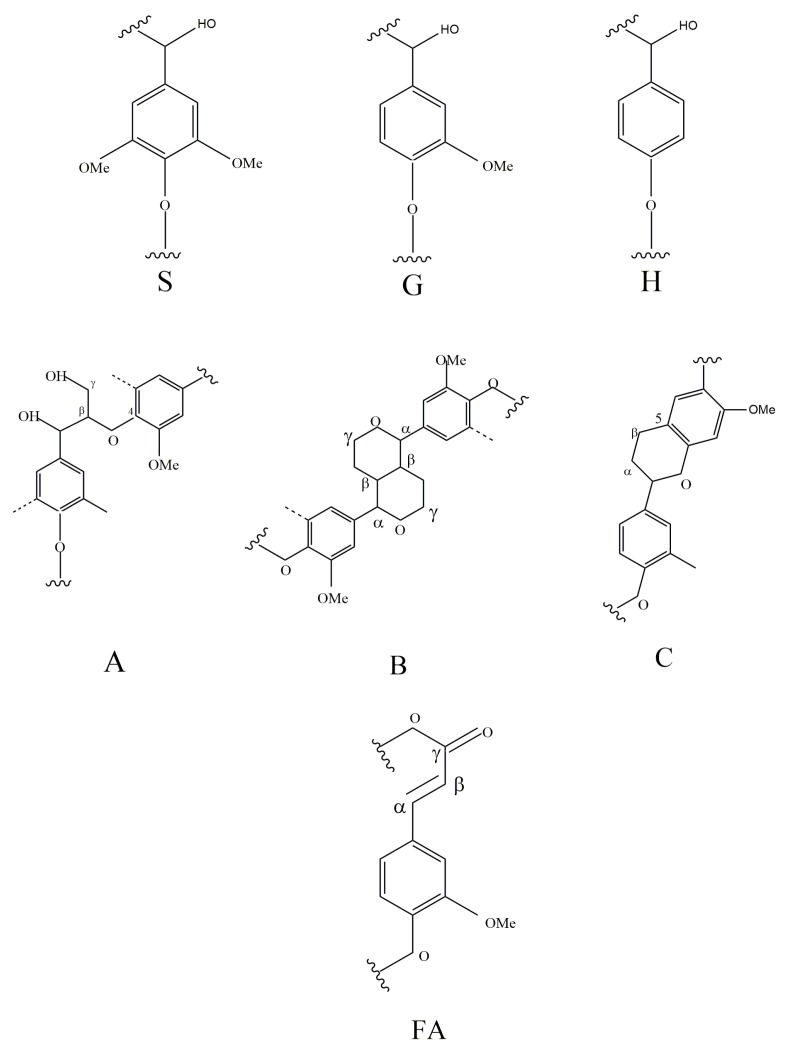
Main classical structures in the lignin preparations: (**A**) β–*O*–4 ether linkages; (**B**) resinol structures formed by β-β’/α-*O*-γ’/γ-*O*-α’ linkages; (**C**) phenylcoumarane structures formed by β-5 ’/α-*O*-4′ linkages; (**FA**) Ferulates guaiacyl unit; (**S**) syringyl unit; (**H**) p-hydroxyphenyl units (**G**) guaiacyl units.

To better characterize lignin structure, 1–6 year-old bamboo lignin samples were further examined by 2D-HSQC spectra, which can indicate the side chain region (δC/δH 50–90/2.5–6.0) and the aromatic ring region (δC/δH 90–160/6.0–8.0). The main structure and signal attribution are compared with the reported data [8,33]. Results are shown in Figure 4 and Table 2.

**Figure 5:**
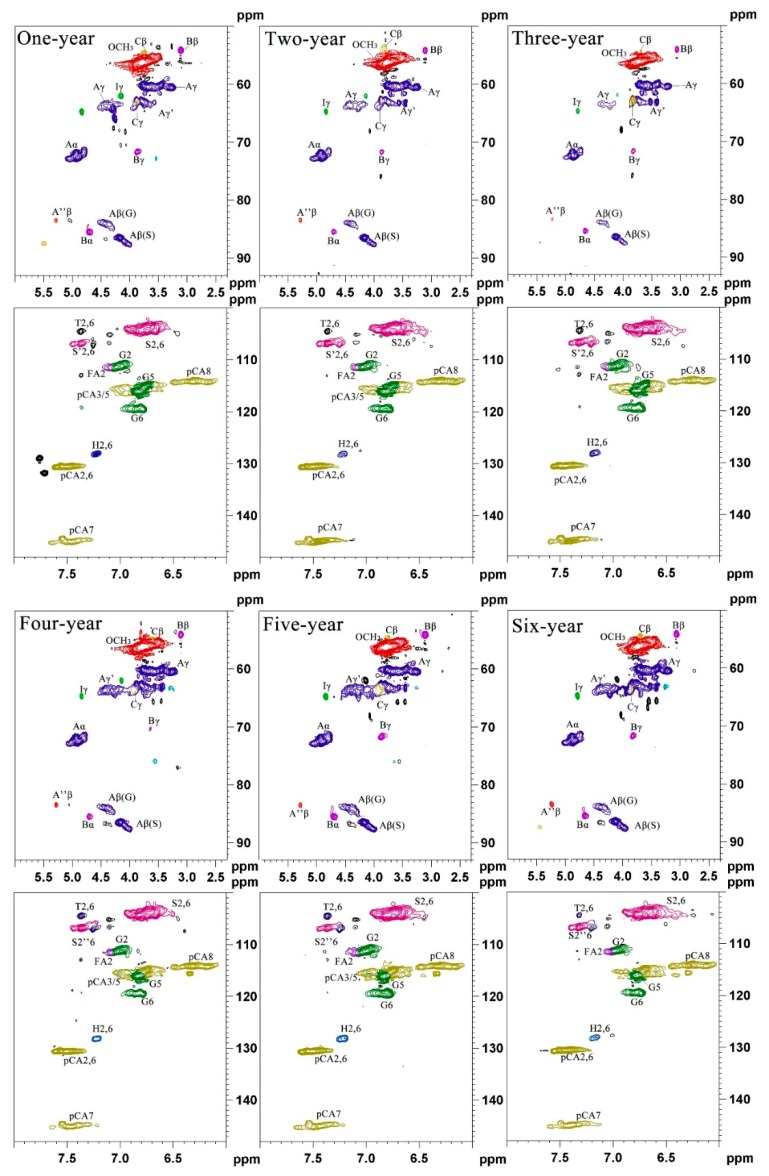
2D-HSQC NMR spectra of 1–6 year-old bamboo lignins.

Signal Aγ-(δ63.0/4.36 ppm) appeared in all bamboo lignin samples, indicating that the γ-position of lignin has undergone acylation during the lignification of bamboo, which was consistent with the literature reports [24]. Three basic units of lignin (S, G, H) can be observed in the aromatic ring region and the side chain region (Figure 5). From a structural point of view, the signs of lignin production in one to six year-old bamboo present little changes. The main signals of syringyl unit are Aβ(S)-thero (δ79.8/4.63 ppm), Aβ(S)-erythro (δ79.8/4.63 ppm), S2,6 (δ103.5/2.66 ppm), S′′2,6 (δ106.3/7.32 ppm). The main signals for the p-hydroxytoluene unit are H2,6 (δ127.8/7.34 ppm), H3,5 (δ115.1/6.52 ppm). The main signals of guaiac wood-based units are Aβ(G) (δ76.6/5.07 ppm), G2 (δ110.8/6.97 ppm), G5 (δ114.5/6.71 ppm), G6 (δ119.0/6.78 ppm). It is proved that the bamboo lignin has three major monomers, which is consistent with the results of FT-IR and ^13^C NMR.

It is worth noting that there are obvious methoxy signal units (δ55.6/3.70 ppm) in the side chain region, which is consistent with the ^13^C NMR result. The main linkage units include β-*O*-4 ether linkages, β-β, and β-5. The β-*O*-4 ether bond has three structures: hydroxyl group (A), acetyl group (A′), and esterified pair bean ester (A′′). The resin alcohol (B) structural unit also appeared as three signals with α, β, and γ positions at δ 85.1/4.66 ppm, δ53.7/3.74 ppm, and δ71.5/4.28 ppm, respectively. All the above connection structure can be detected in 1–6 year-old bamboo lignin, indicating that the connection modes of lignin are similar during the lignification of bamboo.

### 3.6. Semiquantitative Estimations Based on 2D-HSQC Spectra

To provide explicit structural evolution of the 1–6 year-old bamboo lignin samples, semiquantitative of the linkages by 2D-HSQC NMR was performed according to the relevant literature [1,8,32]. It is worth noting that G-type lignin is preferentially located in the middle lamella and S-type lignin mainly situated in the inner cell wall area [34]. Different separation methods result in different content of S-type and G-type lignin. In short, ball-milled lignin tends to contain lignin from the middle lamella, whereas cellulase-digested lignin represents lignin from the secondary cell wall [35]. Therefore, compared to cellulase-digested lignin, the content of G-type lignin in milled bamboo lignin was slightly higher, resulting in a lower S/G ratio. Since the main purpose of this study was to investigate the change of bamboo lignin during lignification process, it was necessary to explore the relative proportion of S/G. As shown in Table 3, S/G ratio has shown an overall upward trend with the bamboo lignification process (1.94–3.02), indicating that the increase rate of S-type lignin content is higher than that of G-type lignin. Therefore, the inner wall area of the cell wall is continuously thickened during the lignification process. This result is consistent with the reported phenomenon by the slice method [19]. Moreover, the deposition of S-type lignin plays an important role in the growth and maturation of angiosperms, which is consistent with the increment of S/G ratio observed in this experiment. The conclusions drawn by the 2D-HSQC semiquantitative method appear to confirm this view. Moreover, the content of β-*O*-4 ether units shows the similar upward trend (84.13–92.95) as the S/G ratio, which means that S-type lignin content increases constantly. This phenomenon is mainly because the β-*O*-4 ether unit is mainly connected with S-type lignin [36]. On the other hand, β-*O*-4 linkage played an important role in angiosperms growth, thus it can act as a pivotal indicator to reflect the lignin complex intactness. The higher the β-*O*-4 linkage content, the more G-type lignin involved in the construction of β-*O*-4 dimers [33]. In contrast, the content of resinol substructures and phenylcoumaran show an opposite downward trend. Resinol substructures are the second most abundant interunit in bamboo lignin, which is mainly connecting with G-type lignin by its available 5-position with monolignol β-positions. Thus, the decrease of resinol substructures may be related to the decrease of G-type lignin [35]. The increment of β-*O*-4 ether unit content is consistent with the GPC results: the Mw of lignin increases with the lignification process. In general, the lower S/G ratio, the more suitable the plant is for the paper-making industry. Lignin will be removed better from bamboo under alkaline conditions with lower S/G ratio, and the fiber strength of the original bamboo will be retained to a greater extent [37]. Therefore, one-year-old bamboo is mainly used in the papermaking industry, while high-grade bamboo is more suitable for the construction field.

## 4. Conclusions

In this paper, changes in bamboo lignin with age (1–6 years old) were investigated. Results showed that the lignin of 1–6 year-old bamboo contained three monomers of S, G and H with similar chemical structure. The molecular weight of bamboo lignin increases with age. 2D-HSQC NMR semiquantitative results further confirmed that the main change of lignin during the lignification process of bamboo occurs in the monomer content: S/G ratio enhanced with the progress of lignification. The main connecting structure of lignin was the β-*O*-4 ether bond, and the relative content of β-*O*-4 ether bond increased during the progress of lignification, which is consistent with the GPC results. Qualitative results showed that the structure of bamboo lignin did not change significantly during the lignification process, while the quantitative results presented that S/G ratio of bamboo lignin increased with age. In the future studies, biological methods such as sectioning and staining observation of cells will be applied to further verify and explore new findings regarding changes of lignin in the bamboo lignification process.

## Figures and Tables

**Table 1:** Weight-average (Mw) and number-average (Mn) molecular weights and polydispersity (Mw/Mn) of 1–6 years old bamboo lignin samples.

Age (Years)	Mw (g/mol)	Mn (g/mol)	Mw/Mn
1	4586	2327	1.97
2	4954	2329	2.12
3	5245	2477	2.12
4	5410	2570	2.11
5	5670	2584	2.19
6	5823	2484	2.34

**Table 2:** Assignments of ^13^C-^1^H cross signals in the HSQC spectra of the lignin.

Label	δC/δH (ppm)	δC/δH (ppm)	Assignments
B′_β_	49.8/2.56	ND	C_β_–H_β_ in β-β tetrahydrofuran (B′)
C_β_	53.1/3.46	ND	C_β_–H_β_ in phenylcoumaran (C)
B_β_	53.5/3.07	53.5/3.07	C_β_–H_β_ in β-β (resinol) (B)
D_β_	59.8/2.75	ND	C_β_–H_β_ in spirodienones (D)
OCH_3_	56.4/3.70	55.6/3.76	C–H in methoxyls
A_γ_	59.9/3.35	62.0/4.08	C_γ_–H_γ_ in β–*O*–4 substructures (A)
A′_γ_	63.0/4.36	63.0/3.8	C_γ_–H_γ_ in γ-acylated β–O–4 (A′)
C_γ_	62.2/3.76	64.3/4.33	C_γ_–H_γ_ in phenylcoumaran (C)
I_γ_	61.2/4.09	64.1/4.66	C_γ_–H_γ_ in cinnamyl alcohol end-groups (I)
I′_γ_	64.0/4.80	64.2/4.82	C_γ_–H_γ_ in acylated cinnamyl alcohol (I′)
B_γ_	71.2/3.82	71.7/3.84	C_γ_–H_γ_ in β-β resinol (B)
A_α_	71.8/4.86	73.8/5.93	C_α_–H_α_ in β–*O*–4 unit (A) *(Erythro)*
A_α_	71.8/4.86	73.8/5.93	C_α_–H_α_ in β–*O*–4 unit (A) *(Thero)*
A_β_(G)	83.4/4.38	76.6/5.07	C_α_–H_α_ in β–*O*–4 linked to G(A)
B_α_	84.8/4.66	85.6/4.70	C_α_–H_α_ in β-β resinol (B)
B′_α_	83.2/4.94	ND	C_α_–H_α_ in β-β (B′, tetrahydrofuran)
A′′_β_	82.8/5.23	ND	C_β_–H_β_ in β–*O*–4 substructures (A)
A′_β_(G)	80.8/4.52	ND	C_β_–H_β_ in acylated β–*O*–4 linked to G (A)
A_β_(S)	85.8/4.12	79.8/4.63	C_β_–H_β_ in β–*O*–4 linked to S (A, *Erythro*)
A_β_(S)	85.8/4.12	79.8/4.63	C_β_–H_β_ in β–*O*–4 linked to S (A, *Thero*)
D_α_	81.0/5.10	ND	C_α_–H_α_ in spirodienones (D)
D′_α_	79.4/4.10	ND	C′_α_–H′_α_ in spirodienones (D)
E_α_	79.6/5.60	ND	C_α_–H_α_ in α,β-diaryl ethers (E)
C_α_	86.8/5.45	87.1/5.49	C_α_–H_α_ in phenylcoumaran (C)
T′_2,6_	103.9/7.34	ND	C′_2,6_–H′_2,6_ in tricin (T)
T_6_	98.9/6.23	ND	C_2,6_–H_2,6_ in tricin (T)
T_8_	94.2/6.60	ND	C_8_–H_8_ in tricin (T)
T_3_	106.2/7.07	ND	C_3_–H_3_ in tricin (T)
S_2,6_	103.9/6.70	103.5/6.66	C_2,6_–H_2,6_ in syringyl units (S)
S′_2,6_	106.3/7.32	105.4/7.37	C_2,6_–H_2,6_ in oxidized S units (S′)
G_2_	110.8/6.97	111.0/7.07	C_2_–H_2_ in guaiacyl units (G)
G_5_	114.5/6.70	116.5/7.00	C_5_–H_5_ in guaiacyl units (G)
G_5e_	115.1/6.95	ND	C_5_–H_5_ in etherified guaiacyl units (G)
G_6_	119.0/6.78	118.9/6.90	C_6_–H_6_ in guaiacyl units (G)
J_β_	126.1/6.76	ND	C_β_–H_β_ in cinnamyl aldehyde end-groups (J)
H_2,6_	127.7/7.17	127.8/7.34	C_2,6_–H_2,6_ in H units (H)
PCE_3,5_	115.6/6.77	122.1/7.14	C_3,5_–H_3,5_ in *p*-coumarate (*p*-CE)
PCE_2,6_	130.2/7.48	129.3/7.68	C_2,6_–H_2,6_ in *p*-coumarate (*p*-CE)
PCE_7_	144.8/7.51	143.5/7.52	C_7_–H_7_ in *p*-coumarate (*p*-CE)
PCE_8_	113.7/6.24	117.4/6.45	C_8_–H_8_ in *p*-coumarate (*p*-CE)
FA_2_	110.7/7.35	ND	C_2_–H_2_ in ferulate (*p*-FA)
FA_6_	123.1/7.20	ND	C_6_–H_6_ in ferulate (*p*-FA)
FA_7_	144.8/7.51	143.5/7.52	C_7_–H_7_ in ferulate (*p*-FA)
J_α_	153.4/7.59	ND	C_α_–H_α_ in cinnamyl aldehyde end-groups (J)

**Table 3:** Quantification of 1–6 year-old bamboo lignin samples by quantitative 2D-HSQC NMR.

	1	2	3	4	5	6
S/G ratio	1.94	1.69	2.25	2.48	3.02	2.92
β-*O*-4 ether units (%)	84.13	86.85	89.85	92.04	92.95	89.15
Resinol substructures(B) (%)	4.37	4.13	2.54	1.81	1.15	2.02
Phenylcoumaran(C) (%)	1.19	1.45	0.57	0.39	0.28	0.36
